# Combination Therapy of Alpha-Lipoic Acid, Gliclazide and Ramipril Protects Against Development of Diabetic Cardiomyopathy *via* Inhibition of TGF-β/Smad Pathway

**DOI:** 10.3389/fphar.2022.850542

**Published:** 2022-03-21

**Authors:** George J. Dugbartey, Quinsker L. Wonje, Karl K. Alornyo, Louis Robertson, Ismaila Adams, Vincent Boima, Samuel D. Mensah

**Affiliations:** ^1^ Department of Pharmacology and Toxicology, School of Pharmacy, College of Health Sciences, University of Ghana, Accra, Ghana; ^2^ Department of Medicine and Therapeutics, University of Ghana Medical School, College of Health Sciences, University of Ghana, Accra, Ghana

**Keywords:** alpha-lipoic acid (ALA), type 2 diabetes mellitus (T2DM), diabetic cardiomyopathy (DCM), anti-diabetic therapy, triple combination therapy

## Abstract

**Background:** Diabetic cardiomyopathy (DCM) is a major long-term complication of diabetes mellitus, accounting for over 20% of annual mortality rate of diabetic patients globally. Although several existing anti-diabetic drugs have improved glycemic status in diabetic patients, prevalence of DCM is still high. This study investigates cardiac effect of alpha-lipoic acid (ALA) supplementation of anti-diabetic therapy in experimental DCM.

**Methods:** Following 12 h of overnight fasting, 44 male Sprague Dawley rats were randomly assigned to two groups of healthy control (*n* = 7) and diabetic (*n* = 37) groups, and fasting blood glucose was measured. Type 2 diabetes mellitus (T2DM) was induced in diabetic group by intraperitoneal (i.p.) administration of nicotinamide (110 mg/kg) and streptozotocin (55 mg/kg). After confirmation of T2DM on day 3, diabetic rats received monotherapies with ALA (60 mg/kg; *n* = 7), gliclazide (15 mg/kg; *n* = 7), ramipril (10 mg/kg; *n* = 7) or combination of the three drugs (*n* = 7) for 6 weeks while untreated diabetic rats received distilled water and were used as diabetic control (*n* = 9). Rats were then sacrificed, and blood, pancreas and heart tissues were harvested for analyses using standard methods.

**Results:** T2DM induction caused pancreatic islet destruction, hyperglycemia, weight loss, high relative heart weight, and development of DCM, which was characterized by myocardial degeneration and vacuolation, cardiac fibrosis, elevated cardiac damage markers (plasma and cardiac creatine kinase-myocardial band, brain natriuretic peptide and cardiac troponin I). Triple combination therapy of ALA, gliclazide and ramipril preserved islet structure, maintained body weight and blood glucose level, and prevented DCM development compared to diabetic control (*p* < 0.001). In addition, the combination therapy markedly reduced plasma levels of inflammatory markers (IL-1β, IL-6 and TNF-α), plasma and cardiac tissue malondialdehyde, triglycerides and total cholesterol while significantly increasing cardiac glutathione and superoxide dismutase activity and high-density lipoprotein-cholesterol compared to diabetic control (*p* < 0.001). Mechanistically, induction of T2DM upregulated cardiac expression of TGF-β1, phosphorylated Smad2 and Smad3 proteins, which were downregulated following triple combination therapy (*p* < 0.001).

**Conclusion:** Triple combination therapy of ALA, gliclazide and ramipril prevented DCM development by inhibiting TGF-β1/Smad pathway. Our findings can be extrapolated to the human heart, which would provide effective additional pharmacological therapy against DCM in T2DM patients.

## 1 Introduction

Diabetic cardiomyopathy (DCM) is a common and major long-term cardiovascular complication of diabetes mellitus, which involves structural and myocardial dysfunction independent of traditional risk factors such as hypertension, coronary artery disease or other cardiac diseases (Yang et al., 2018; Ge et al., 2019). It is more common among patients with type 2 diabetes mellitus (T2DM) than their counterparts with type 1 diabetes mellitus (T1DM) due to the increased incidence of T2DM than T1DM among the diabetic population (Robillon et al., 1994; Xu et al., 2018). Considering the high global prevalence of diabetes mellitus, DCM has become a major public health problem worldwide. It accounts for over 20% of annual mortality rate of diabetic patients globally (Tillquist and Maddox, 2012), with significantly higher hospitalization for heart failure compared to nondiabetic patients with heart failure (Lee C. et al., 2012; Lindman et al., 2014). In addition, it is reported that diabetic patients have up to a 74% increased risk of developing DCM, and those with DCM are four times more likely to die than those without DCM (Holman et al., 2021). Human post-mortem and animal studies show that DCM clinically presents with cardiac hypertrophy, pathological alterations in ventricular structure, increased interstitial and perivascular fibrosis as well as diastolic and systolic dysfunction with preserved ejection fraction, which is accompanied by reduced cardiomyocyte contraction and changes in specific cardiomyocyte proteins (Rubler et al., 1972; Penpargkul et al., 1981; Trost et al., 2002).

Although several existing anti-diabetic therapies have been successful in lowering hyperglycemia in T2DM, the high prevalence of DCM still persists among these patients. This suggests that additional factors beyond hyperglycemia contributes to the development and progression of DCM in diabetic patients. A 5-year clinical study from 1998 to 2003 showed that oxidative stress due to overproduction of reactive oxygen species (ROS; a destructive mediator of tissue injury) is a principal cause of cardiomyocyte apoptosis in DCM, which is accompanied by reduced antioxidant status (Kuethe et al., 2007). Hence, in addition to persistent hyperglycemia and ROS-induced oxidative stress, a number of complex and interrelated molecular mechanisms have been identified to underlie the development and progression of DCM. These mechanisms have been reviewed recently to include impaired insulin metabolic signaling in diabetic cardiomyocytes, increased free fatty acid production, mitochondrial dysfunction, increased advanced glycation end-products, inflammation, inappropriate activation of renin-angiotensin-aldosterone system, endoplasmic reticulum stress and impaired cardiomyocyte calcium handling (Jia et al., 2016; Jia et al., 2018). Among these mechanisms, ROS-induced oxidative stress has received significant experimental attention as the unifying contributing factor in DCM development and progression (Kakkar et al., 1995; Ghosh et al., 2004; Hamblin et al., 2007; Singh et al., 2008; Das et al., 2012; Tocchetti et al., 2015). Burgeoning evidence also shows that ROS-induced oxidative stress contributes to development of cardiac fibrosis in DCM by upregulating the expression of transforming growth factor-beta 1 (TGF-β1; a central mediator of cardiac fibrosis) (Purnomo et al., 2013; Zhang et al., 2018; Wang et al., 2019; Wang et al., 2021). TGF-β1 induces remodeling and fibrosis in cardiac tissue and other tissues through activation of its downstream effector proteins referred to as Smads, which include Smad 2/3, Smad 4 and other Smads. As transcription factors, Smads form complexes following activation by TGF-β1, and mediate nuclear translocation and gene transcription, which subsequently lead to proliferation and synthesis of extracellular matrix in human cardiac fibroblasts under diabetic and other pathological conditions, leading to myocardial fibrosis (Song et al., 2013; Zeglinski et al., 2015; Zhang Y. et al., 2018). Unfortunately, although current pharmacological agents provide some reduction in oxidative stress in DCM, there is no consensus on their clinical outcomes, as global prevalence of DCM is still high. Therefore, there is urgent need to identify novel effective antioxidant therapy in combination with conventional anti-diabetic therapy to specifically target ROS-induced oxidative stress and other pathophysiological pathways including TGF-β1/Smad pathway in DCM.

Consistent with the above premise, alpha-lipoic acid (ALA), a disulphide compound and a natural antioxidant, which functions as an essential cofactor for several mitochondrial enzymes in glucose oxidation and ATP generation, has been suggested to be beneficial in diabetes and in a number of diabetic complications. It is synthesized in the mitochondria by lipoic acid synthase in cardiomyocytes and other cell types (Mayr et al., 2014). In addition, it is also obtained from plant and animal sources and can also be given as a dietary supplement (Shay et al., 2009). A growing body of preclinical and clinical evidence shows that ALA effectively improves glucose homeostasis and lipid profile in both T1DM and T2DM and also plays a cardioprotective role against development and/or progression of DCM through its potent multifunctional antioxidant property (Strödter et al., 1995; Midaoui et al., 2003; Li et al., 2009; Lee et al., 2012; Li et al., 2012; Lee et al., 2017; Akbari et al., 2018; Târtea et al., 2020). It is also beneficial in clinical and experimental models of other diabetic complications such as diabetic peripheral neuropathy (Lee T. H. et al., 2012; Ziegler et al., 2016; Verma, 2018), diabetic nephropathy (Chang et al., 2007; Lee et al., 2009) and gestational diabetes mellitus (Aslfalah et al., 2019). Furthermore, ALA has also been reported to exert anti-inflammatory effect in different animal models of cardiac injury (Sridharan et al., 2017; Shen et al., 2019) and other organs (Mervaala et al., 2003). Therefore, we hypothesized that a triple combination therapy of ALA, gliclazide (a commonly used anti-diabetic drug) and ramipril (a cardioprotective and blood pressure-control agent) could increase myocardial antioxidant status and suppress cardiac fibrosis via inhibition of TGF-β1/Smad pathway in a rat model of DCM in T2DM.

## 2 Materials and Methods

### 2.1 Drugs and Chemicals

Nicotinamide (Nanjing Yasnt Bio-Tech Co. Ltd.—Nanjing, China), streptozotocin (STZ; Sigma Aldrich, Missouri, United States), gliclazide (Servier Laboratories Limited, France), alpha-lipoic acid (ALA; Nanjing Yasnt Bio-Tech Co. Ltd.—Nanjing, China), ramipril (Teva Pharmaceuticals, United Kingdom). TGF-β1 primary antibody (cat. no. SC7892, Santa Cruz Biotechnology, Inc.), phospho-Smad2 (cat. no. ab53100, Abcam, Canada), phospho-Smad3 (cat. no. ab52903, Abcam), β-actin (cat. no. ab8227, Abcam, Canada), horseradish peroxidase (HRP)-conjugated secondary antibodies (cat. no. ab13168, Abcam; cat. nos. sc-2350 and sc-2371, Santa Cruz Biotechnology, Inc.).

### 2.2 Ethical Statement

The study protocol was approved by the Institutional Animal Care and Use Committee of the University of Ghana. The experiment was performed in the Laboratory Animal Facility (with Office of Laboratory Animal Welfare assurance number A7604-01) of the Noguchi Memorial Institute for medical research according to the experimental protocol while maintaining quality assurance in accordance with good laboratory practice. All procedures and techniques used in this study were in accordance with the National Institute of Health Guidelines for the care and use of laboratory animals.

### 2.3 Animal Handling

Forty-four male Sprague-Dawley rats weighing 150–200 g and between 6 and 8 weeks old were obtained from the Department of Animal Experimentation, Noguchi Memorial Institute for Medical Research, University of Ghana, Legon, Accra. The rats were housed in standard cages in the same Department at 23 ± 2°C ambient temperature and relative humidity of 45–55% at a 12:12 h light:dark cycle. They were fed with standard rat chow (Agricare, Kumasi) and tap water *ad libitum* and allowed to acclimatize for 7 days prior to the start of the experiment.

### 2.4 Animal Experimental Protocol

#### 2.4.1 Establishment of T2DM Animal Model

Following acclimatization and 12 h of overnight fasting, fasting blood glucose (FBG) was measured with a portable hand-held glucometer (One Touch Select Plus®; LifeScan Inc., Zug, Switzerland), and rats were randomly assigned to two groups of healthy control (*n* = 7) and diabetic groups (*n* = 37). T2DM was induced in the diabetic group by intraperitoneal (i.p.) administration of 110 mg/kg nicotinamide followed by i.p. injection of freshly prepared 55 mg/kg streptozotocin (STZ; dissolved in freshly prepared 0.1 M citrate buffer of pH 4.5) as described previously (Brahmanaidu et al., 2017; Phatak et al., 2019). FBG was measured after 3 days of T2DM induction, and rats with FBG values above 13.9 mmol/L (250 mg/dl) were considered diabetic and included in this study as previously described (Phatak et al., 2019).

#### 2.4.2 Treatment of Diabetic Animals

Upon confirmation of T2DM on day 3, the diabetic rats received either oral daily administration of 15 mg/kg gliclazide (DM + GLC group; *n* = 7), 60 mg/kg alpha-lipoic acid (DM + ALA group; *n* = 7), 10 mg/kg ramipril (DM + RAM group; *n* = 7) or combinations of these drugs (DM + ALA + GLC + RAM group; *n* = 7 using the same individual doses) for 6 weeks while another group of diabetic rats (DM Untreated; *n* = 9) received distilled water (100 g/kg per day) and served as diabetic control. Body weights and glycosylated hemoglobin A1c (HbA1c) levels were measured on days 7, 14, 21, 28, 35 and 42 using an automatic biochemical analyzer (Nycocard Reader, Axis Shield, Oslo, Norway) at Tema General Hospital, Ghana. The measurement of HbA1c is based on immunoturbidimetric determination of the stable glucose adduct to the N-terminal group of hemoglobin β chain.

#### 2.4.3 Euthanasia and Organ Harvest

After 6 weeks of treatment, all groups of rats were euthanized with ketamine:xylazine intraperitoneally. About 8 ml of blood sample was obtained from each rat *via* cardiac puncture and transferred into EDTA and Eppendorf tubes for biochemical analysis. Heart and pancreas were harvested and weighed. Mid-ventricular heart sections were isolated, and together with the pancreas, were stored in 10% neutral buffered formalin for histological analysis, while the apical sections of the heart were snap-frozen in liquid nitrogen and transferred into a −80°C freezer for molecular and other analyses. Relative heart weight (heart weight/body weight ratio) was calculated by expressing the weight of each heart as a percentage of the rat’s body weight.

### 2.5 Plasma Preparation and Biochemical Analysis

Blood samples in EDTA tubes were centrifuged at 3,000 rpm for 15 min at 4°C. Plasma samples obtained from centrifugation were stored in Eppendorf tubes at −20°C. Plasma levels of total cholesterol, triglycerides, high-density lipoproteins-cholesterol (HDL) were measured by an automatic biochemical analyzer at the Tema General Hospital, Ghana, and according to the manufacturer’s instructions (Mindray BS-200 Biochemistry Auto-analyzer, Shenzhen, China) as previously described (N’guessan et al., 2021). Plasma levels of tumor necrosis factor-alpha (TNF-α), interleukin-1β and interleukin-6 (IL-1β and IL-6; inflammatory markers) were also measured by ELISA as previously described (Bortolon et al., 2012) using a DuoSet Kit according to the manufacturer’s instructions (Quantikine, R&D Systems, Minneapolis, MN, United States). Also, plasma levels of cardiac damage markers such as creatine kinase-myocardial band (CK-MB) and brain natriuretic peptide (BNP) were measured using an RA 50 semi-auto analyzer.

### 2.6 Tissue Processing and Histological Examination

Midventricular heart sections and pancreas tissue samples were processed for histological examination as previously described (Arow et al., 2020) with some modifications. Briefly, after fixation with 10% neutral buffered formalin, the middle piece of the heart (ventricles) and pancreas were dehydrated in an increasing order of alcohol concentration (70, 80, 90 and 100%) followed by dehydration in xylene and finally embedded in molten paraffin wax. The paraffin-embedded tissues were sectioned at 4 μm-thick. The sections were dried in an oven at 60°C for 3 h. Next, they were deparaffinised in xylene and hydrated in decreasing series of alcohol (100, 95, 80%) and then in distilled water. The sections were stained with hematoxylin-eosin stain and periodic acid Schiff (PAS) stain. The stained tissue sections were examined under light microscope and the images were captured with a digital camera attached to it. The histological sections of the heart were scored independently and in a double-blinded fashion by two experienced pathologists at 400× magnification based on the degree of myocardial degeneration and cardiomyocyte vacuolation as previously described (Moriyama et al., 2010; Atta et al., 2018).

### 2.7 Determination of Antioxidant Status

#### 2.7.1 Measurement of Tissue Glutathione Level and Superoxide Dismutase Activity

Frozen-kept heart tissue samples (50 mg) of each group was tested for glutathione (GSH) content and SOD activity according to the test kit instructions (Nanjing Kaiji Bio, Nanjing, China) and as previously reported by Xu et al. (2019).

#### 2.7.2 Measurement of Malondiadehyde

The levels of malondialdehyde (MDA; a by-product of lipid peroxidation and indicator of ROS-induced oxidative stress) in the heart tissue was measured using thiobarbituric acid reactive substances (TBARS) method as we previously described (Dugbartey et al., 2015). Briefly, about 50 mg of heart tissues were homogenized in 100 ml PBS containing butylated hydroxytoluene (Cell Biolabs, Netherlands) and centrifuged at 10,000 g for 5 min at 4°C. Next, SDS-Lysis solution (50 ml, Cell Biolabs) was added to 50 ml of supernatant and MDA standards, and incubated at room temperature for 5 min. A volume of 125 ml of TBA reagent (Cell Biolabs) was then added and incubated at 95°C for 60 min, followed by cooling to room temperature for 5 min and centrifuged at 1,000 g for 15 min 4°C and the supernatants collected. 2-Butanol (150 ml, Merck, Darmstadt, Germany) was added to supernatants, vortexed for 2 min and centrifuged for 5 min at 10,000 g 4°C. Lipid peroxidation was calculated by measuring optical density at 532 nm in 200 ml of the butanol fraction and expressed as nmol/mg of heart tissue. The TBARS method was repeated using plasma samples and lipid peroxidation was measured at the same optical density of 532 nm.

### 2.8 ELISA for Plasma Cardiac Troponin I

Plasma levels of cardiac troponin I (cTnI; a specific cardiac damage marker) were measured using a commercially available sandwich ELISA kit (High Sensitivity Rat Cardiac Troponin-I ELISA Kit, Life Diagnostics Inc., PA, United States). The reagents in the ELISA kit were incubated at room temperature for 30 min prior to use, and according to the manufacturer’s protocol. Briefly, 50 μL of standard and diluted plasma were added into the respective wells of plates. Subsequently, 100 μL of enzyme-linked reagents were added into each well and then incubated at 37°C for 60 min. The mixture in each well was discarded and the developing agents were added. Next, the plates were placed in the dark for 20 min and the absorbance of each well was determined at 450 nm using a microplate reader (RT 6100; Rayto Life and Analytical Sciences Co., Ltd., Shenzhen, China).

### 2.9 Western Blotting

About 50 mg of frozen-kept cardiac tissue samples were homogenized with RIPA lysis buffer system (Santa Cruz Biotechnology, Inc., Dallas, TX, United States) and phenylmethyl-sulfonylfluoride (PMSF; Santa Cruz Biotechnology, Inc.) according to the manufacturer’s instructions. The homogenate was centrifuged (12,500 × g) at 4°C for 10 min, and the supernatants were collected. Bicinchoninic acid (BCA) protein assay was performed to determined total protein concentration using BCA assay kit (Santa Cruz Biotechnology, Inc.), after which the proteins were subjected to 8% SDS PAGE, and then separated by vertical electrophoresis. Next, the protein samples were transferred to polyvinylidene fluoride (PVDF) membranes (EMD Millipore, Billerica, MA, United States) electrically at 10 12 V for 50 min. The PVDF membranes were then incubated in specific primary antibodies directed against TGF β1 (cat. no. SC7892, 1:1,000; Santa Cruz, Biotechnology, Inc.), phospho-Smad2 (cat. no. ab53100, 1:2000, Abcam) and phosphor-Smad3 (cat. no. ab52903, 1:2000, Abcam) overnight at 4°C. Following 20 min of washing, the membranes were then incubated in corresponding horseradish peroxidase (HRP) conjugated secondary antibodies (cat. no. ab13168, 1:1,000; Abcam) (cat. nos. sc-2350 and sc-2371, 1:1,000; Santa Cruz Biotechnology, Inc.) for 1 h at room temperature. Protein bands were developed, visualized and quantified using Gene Genome/Gene Tools (Westburg, Leusden, Netherlands). β-actin (cat. no. SC7892, 1:5,000, Santa Cruz Biotechnology, Inc.) was used as a house-keeping protein.

### 2.10 Statistical Analysis

Data are expressed as mean ± standard error of the mean (SEM). Statistical analysis was evaluated using one-way analysis of variance (ANOVA) followed by Tukey’s post-hoc test using Prism software (Prism 8, GraphPad Software, Inc., San Diego, CA, United States). A *p*-value of less than 0.05 between groups is considered statistically significant.

## 3 Results

### 3.1 Effect of Triple Combination Therapy on Body Weight, Blood Glucose Level and Pancreas Structure

**FIGURE 1:**
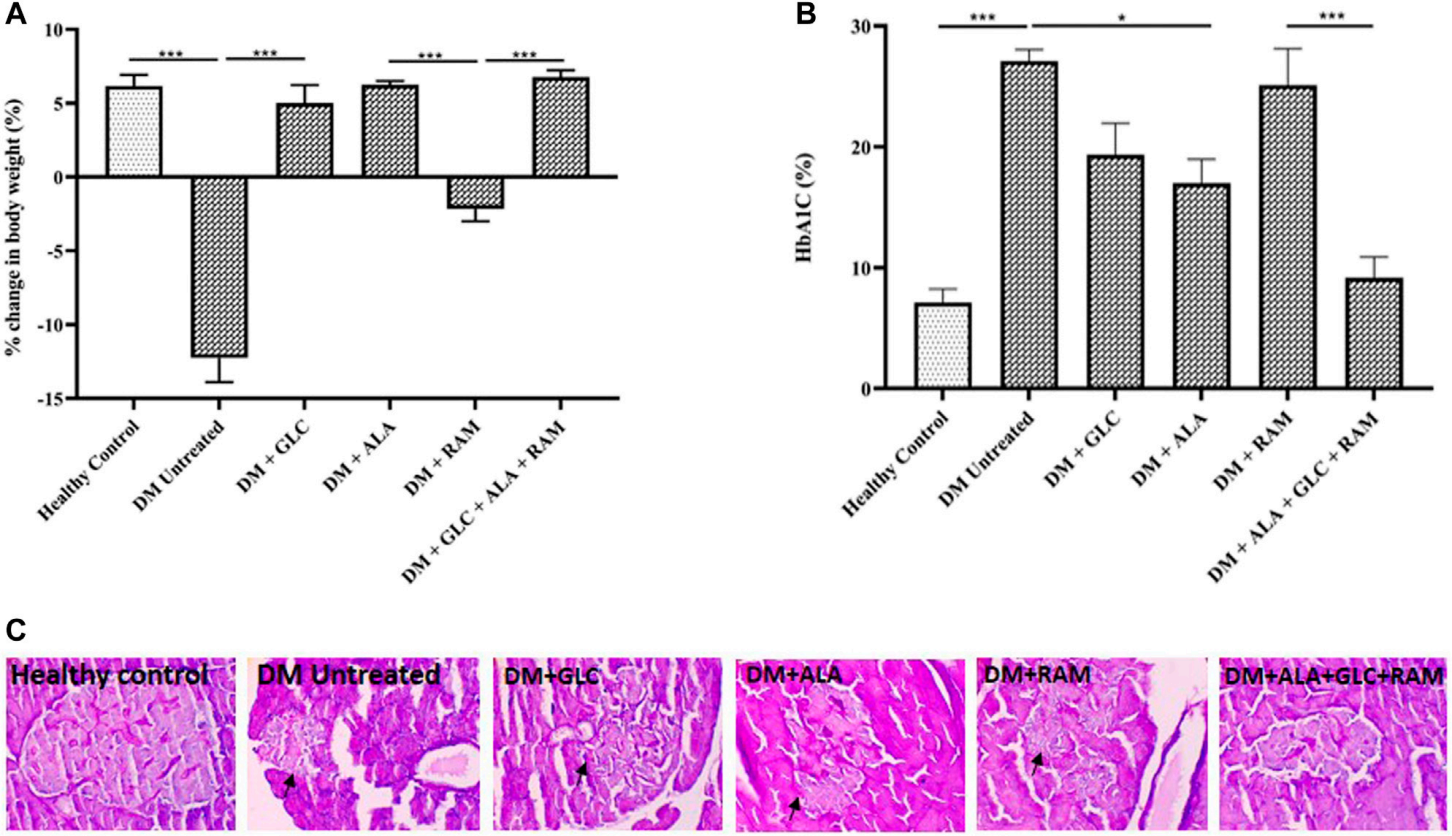
Changes in body weight, HbA1c level and pancreas histology. **(A)** Percentage change in body weight, **(B)** HbA1c level and **(C)** representative photomicrographs of PAS-stained images of pancreatic tissues from all groups. Arrows point to damaged pancreatic islet. Magnification ×400. Healthy control (*n* = 7); DM Untreated = Untreated diabetes mellitus rats (*n* = 9); DM + GLC = Diabetic rats treated with gliclazide (*n* = 7); DM + ALA = Diabetic rats treated with alpha-lipoic acid (*n* = 7); DM + RAM = Diabetic rats treated with ramipril (*n* = 7); DM + ALA + GLC + RAM = Diabetic rats treated with alpha-lipoic acid, gliclazide and ramipril (*n* = 7). *p < 0.05 vs. diabetic control, **p < 0.01 vs. diabetic control, ***p < 0.001 vs. diabetic control.

To determine the effect of triple combination therapy on body weight, blood glucose level and pancreatic islets, we measured the body weight and glycosylated haemoglobin (a reliable marker of blood glucose level) of healthy control and diabetic animals prior to and after induction of T2DM, and also performed histology of the pancreas. Induction of T2DM caused a significant weight loss and destruction of pancreatic islets, which affected insulin-producing β-cells and resulted in hyperglycemia compared to healthy control group (Figures 1A–C; *p* < 0.001). However, with the exception of ramipril group (DM + RAM), monotherapies of diabetic animals with gliclazide (DM + GLC) and alpha-lipoic acid (DM + ALA) and triple combination therapy (ALA + GLC + RAM) prevented the change in body weight (Figure 1A; *p* < 0.001). While monotherpies with GLC and ALA reduced hyperglycemia in the diabetic animals relative to untreated diabetic animals (Figure 1B; *p* < 0.05), monotherapy with ramipril (DM + RAM) had no significant effect on blood glucose level compared to untreated diabetic control group (Figure 1B; *p* > 0.05). However, the best anti-hyperglycemic effect was observed following triple combination therapy, in which normoglycemia was maintained in comparison with healthy control animals (Figure 1B; *p* > 0.05), which translates into better islet protection (Figure 1C). Thus, triple combination therapy with ALA, GLC and RAM maintained body weight and prevented pancreatic islet destruction in diabetic animals, resulting in normoglycemia.

### 3.2 Effect of Triple Combination Therapy on Development of Diabetic Cardiomyopathy and Lipid Profile

**FIGURE 2:**
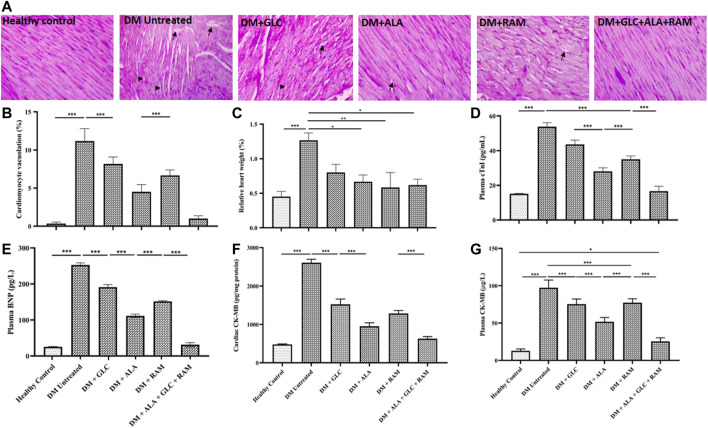
Cardiac histology and damage markers **(A)** Representative photomicrograph of PAS-stained cardiac tissue from all groups. Arrows indicate cardiomyocyte vacuolation and arrow heads indicate myocardial degeneration. ×400 magnification in PAS staining. **(B)** Quantification of cardiomyocyte vacuolation, **(C)** relative heart weight, **(D)** plasma cardiac troponin I (cTnI), **(E)** plasma brain natriuretic peptide (BNP), **(F)** cardiac creatine kinase myoglobin band (CK-MB) and **(G)** plasma creatine kinase myoglobin band (CK-MB). Healthy control (*n* = 7); DM Untreated = Untreated diabetes mellitus rats (*n* = 9); DM + GLC = Diabetic rats treated with gliclazide (*n* = 7); DM + ALA = Diabetic rats treated with alpha-lipoic acid (*n* = 7); DM + RAM = Diabetic rats treated with ramipril (*n* = 7); DM + ALA + GLC + RAM = Diabetic rats treated with alpha-lipoic acid, gliclazide and ramipril (*n* = 7). *p < 0.05 vs. diabetic control, **p < 0.01 vs. diabetic control, ***p < 0.001 vs. diabetic control.

**FIGURE 3:**
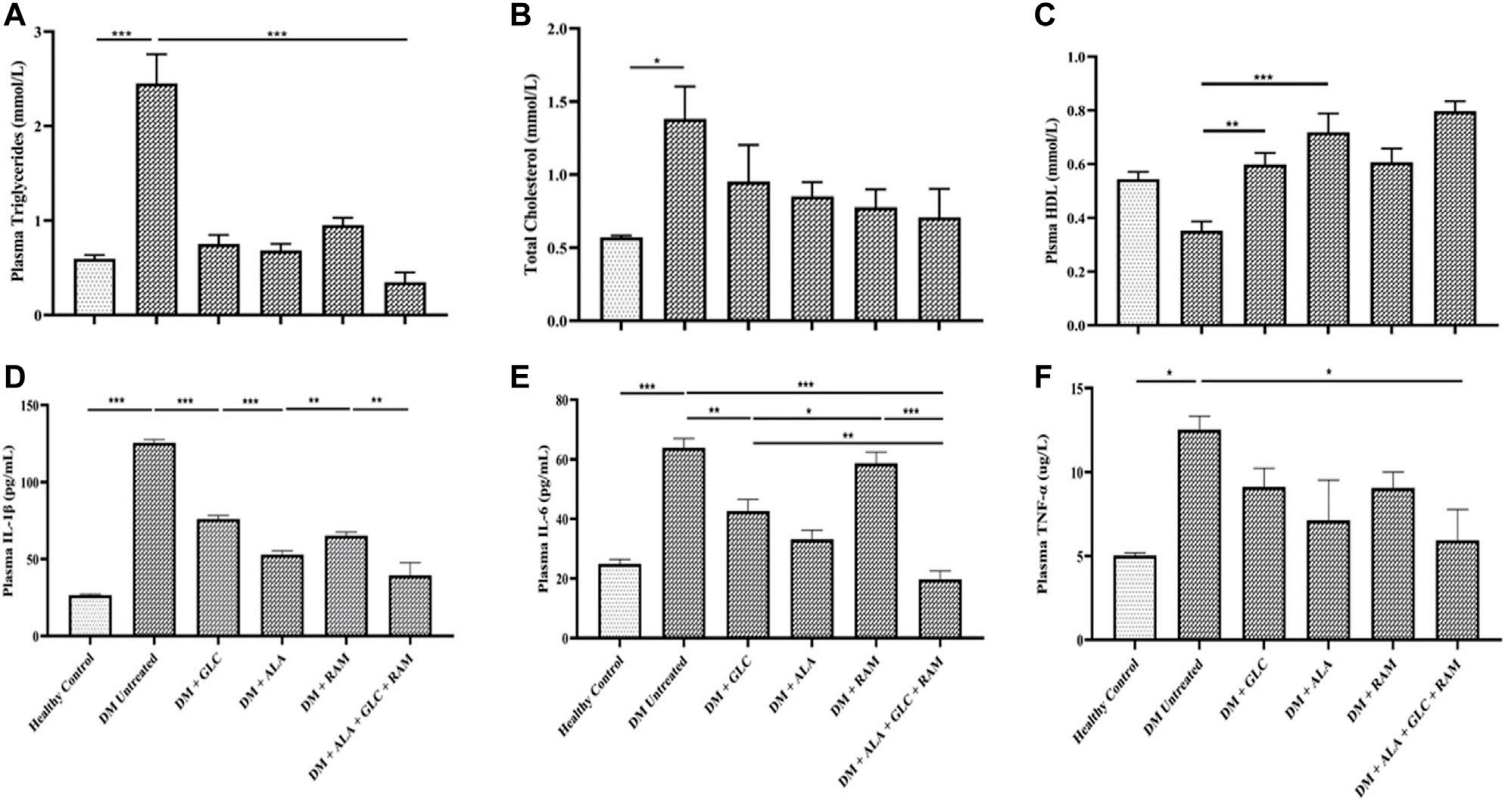
Lipid profile and inflammation. Levels of **(A)** plasma plasma triglycerides, **(B)** total cholesterol, **(C)** plasma high-density lipoprotein (HDL), **(D)** plasma interleukin-1beta (IL-1β), **(E)** plasma interleukin-6 (IL-6) and **(F)** plasma tumor necrosis factor-alpha (TNF-α). Healthy control (*n* = 7); DM Untreated = Untreated diabetes mellitus rats (*n* = 9); DM + GLC = Diabetic rats treated with gliclazide (*n* = 7); DM + ALA = Diabetic rats treated with alpha-lipoic acid (*n* = 7); DM + RAM = Diabetic rats treated with ramipril (*n* = 7); DM + ALA + GLC + RAM = Diabetic rats treated with alpha-lipoic acid, gliclazide and ramipril (*n* = 7). *p < 0.05 vs. diabetic control, **p < 0.01 vs. diabetic control, ***p < 0.001 vs. diabetic control.

To confirm development of diabetic cardiomyopathy, we performed cardiac histology and examined pathological changes following 6 weeks of T2DM induction and treatment and also measured cardiac damage markers in addition to relative heart weight (heart weight/body weight ratio). While heart tissues from healthy control rats showed normal histology characterized by intact myocardial fiber structure and architecture without degeneration and cardiomyocyte vacuolation, heart tissues from untreated diabetic rats revealed marked degeneration and vacuolation of myocardial fibers (Figures 2A,B; *p* < 0.001). Interestingly, cardiac tissues of rats that received monotherapies showed small areas of slight myocardial degeneration and vacuolation compared to untreated diabetic rats (Figures 2A,B; *p* < 0.001) while those from rats that received triple combination therapy showed normal histology similar to healthy control group (Figures 2A,B; *p* > 0.05). The significant pathological alterations in the heart tissue of untreated diabetic rats, suggestive of abnormal heart mass, along with the substantial loss of body weight resulted in markedly higher relative heart weight compared to healthy control and treated rats (Figure 2C; *p* < 0.05). Consistent with the histopathological result, untreated T2DM resulted in significantly elevated cardiac troponin I, brain natriuretic peptide and creatinine kinase myoglobin band in plasma and heart tissue compared to healthy control (Figures 2D–G; *p* < 0.001). Although monotherapies reduced the levels of these cardiac damage markers relative to untreated diabetic group (Figures 2D–G; *p* < 0.05), triple combination therapy remarkably reduced the levels of these biomarkers to levels within the range of healthy control group (Figures 2D–G; *p* > 0.05). In addition, triple combination therapy prevented significant increase in the levels of plasma triglycerides and total cholesterol in diabetic animals (Figures 3A,B; *p* < 0.001) and interestingly increased plasma high-density lipoprotein level beyond that of healthy control group (Figure 3C; *p* < 0.05). Collectively, these results show that triple combination therapy provides the most effective protection against diabetic cardiomyopathy.

### 3.3 Effect of Triple Combination Therapy on Inflammation, Cardiac Oxidative Stress and Fibrosis

**FIGURE 4:**
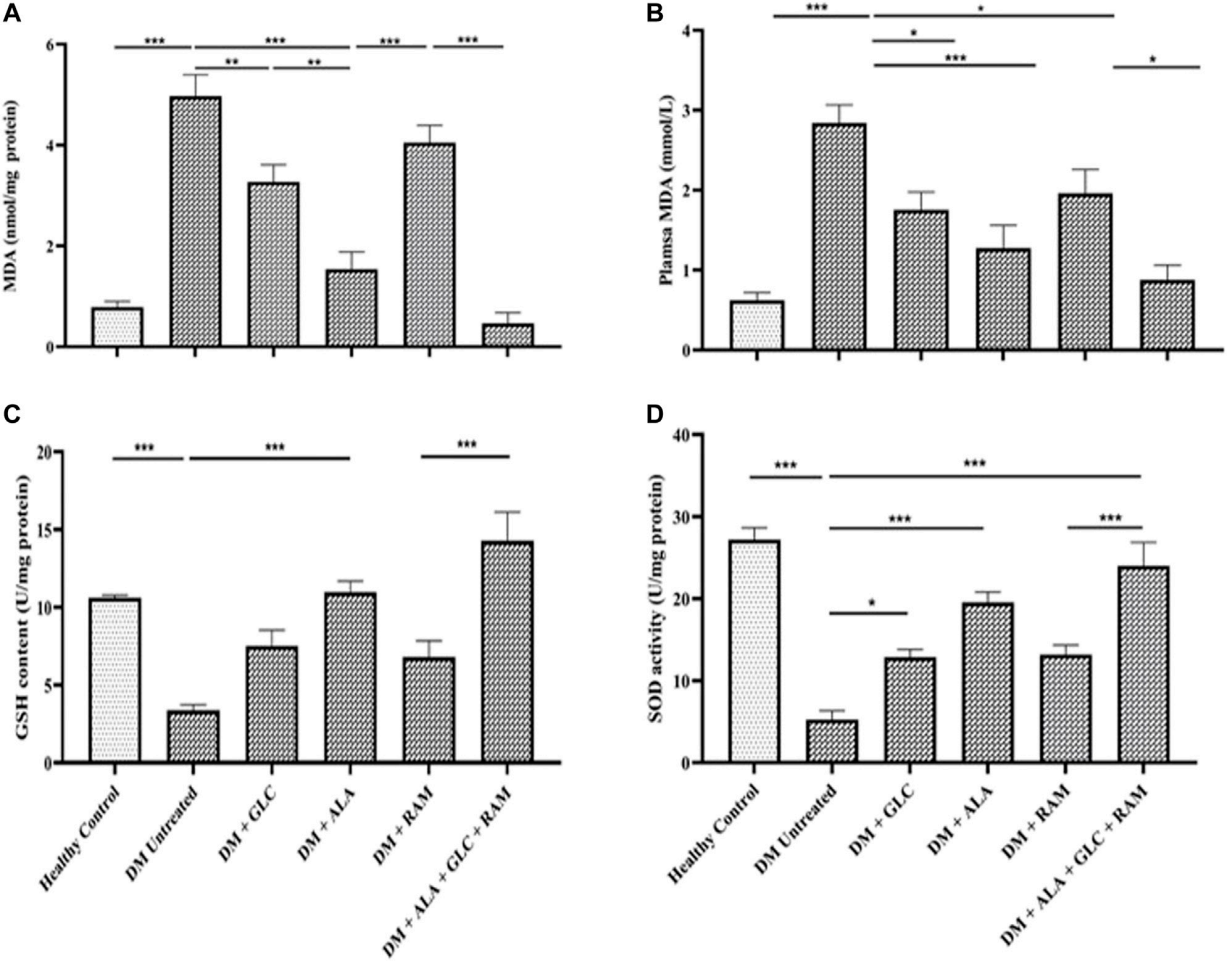
Cardiac antioxidant status. Levels of **(A)** cardiac tissue malondialdehyde (MDA), **(B)** plasma malondialdehyde (MDA), **(C)** cardiac glutathione (GSH) content and **(D)** cardiac superoxide dismutase (SOD) activity. Healthy control (*n* = 7); DM Untreated = Untreated diabetes mellitus rats (*n* = 9); DM + GLC = Diabetic rats treated with gliclazide (*n* = 7); DM + ALA = Diabetic rats treated with alpha-lipoic acid (*n* = 7); DM + RAM = Diabetic rats treated with ramipril (*n* = 7); DM + ALA + GLC + RAM = Diabetic rats treated with alpha-lipoic acid, gliclazide and ramipril (*n* = 7). *p < 0.05 vs. diabetic control, **p < 0.01 vs. diabetic control, ***p < 0.001 vs. diabetic control.

**FIGURE 5:**
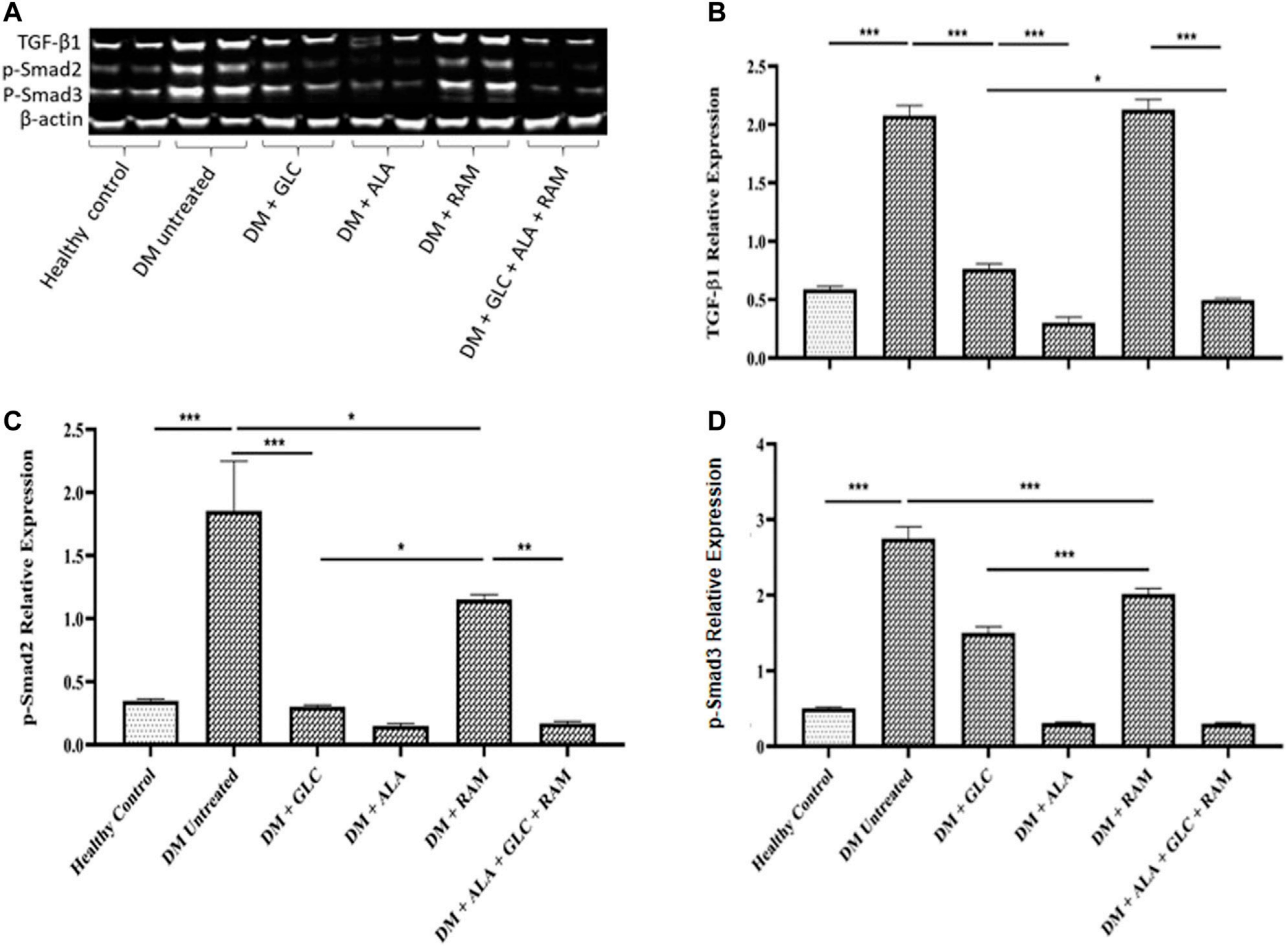
Cardiac fibrosis showing **(A)** Western blot image, and quantification of **(B)** transforming growth factor-beta 1 (TGF-β1), **(C)** phosphorylated Smad2 and **(D)** phosphorylated Smad3. Healthy control (*n* = 7); DM Untreated = Untreated diabetes mellitus rats (*n* = 9); DM + GLC = Diabetic rats treated with gliclazide (*n* = 7); DM + ALA = Diabetic rats treated with alpha-lipoic acid (*n* = 7); DM + RAM = Diabetic rats treated with ramipril (*n* = 7); DM + ALA + GLC + RAM = Diabetic rats treated with alpha-lipoic acid, gliclazide and ramipril (*n* = 7). *p < 0.05 vs. diabetic control, **p < 0.01 vs. diabetic control, ***p < 0.001 vs. diabetic control.

To determine the mechanisms of cardioprotection by the triple combination therapy, we measured plasma levels of pro-inflammatory cytokines (IL-1β, IL-6 and TNF-α), antioxidant status (MDA, GSH and SOD) and fibrosis (TGF-β1/Smad pahway) in the heart tissue. The levels of plasma interleukin-1beta (IL-1β), interleukin-6 (IL-6) and tumor necrosis factor-alpha (TNF-α) were markedly elevated in untreated diabetic control rats, which suggests inflammation compared to those in healthy control rats (Figures 3D–F; *p* < 0.001). Whereas monotherapies showed some cardioprotection by reducing the levels of these pro-inflammatory cytokines in comparison with untreated diabetic rats (Figures 3D–F; *p* < 0.05), triple combination therapy reduced the levels of these cytokines substantially to healthy control levels (Figures 3D–F). A similar pattern was observed in plasma and tissue levels of malondialdehyde (MDA; an indicator of ROS-induced oxidative stress) (Figures 4A,B) while tissue glutathione (GSH) level and superoxide dismutase (SOD) activity in diabetic rats that received the triple combination therapy were markedly increased to healthy control levels (Figures 4C,D), implying decreased oxidative stress and increased antioxidant status in the heart. Also, as shown in Figure 5, the expression of transforming growth factor-1beta (TGF-β1) as well as phosphorylated Smad2 and 3 proteins were significantly upregulated in the heart of untreated diabetic rats, suggesting aggravated cardiac fibrosis compared to healthy control group (Figures 5A–D; *p* < 0.001). Monotherapies significantly reduced the expression levels of these fibrotic proteins relative to untreated diabetic group (Figures 5A–D; *p* < 0.001) albeit not to healthy control level while triple combination therapy strongly downregulated the expression of these proteins to levels slightly lower than that in healthy control levels (Figures 5A–D; *p* > 0.05). Taken together, triple combination therapy with ALA, GLC and RAM and strongly reduced inflammation, oxidative stress and fibrosis in the diabetic heart.

## 4 Discussion

The present study establishes a novel pharmacotherapeutic approach to effectively treating or preventing the development of diabetic cardiomyopathy (DCM) in type 2 diabetes mellitus (T2DM). Using this approach, we show that triple combination therapy of alpha-lipoic acid (ALA), gliclazide and ramipril maintained normoglycemia, preserved myocardial fiber architecture, significantly improved cardiac antioxidant status and inhibited inflammation and cardiac fibrosis, all of which prevented the development of DCM compared to monotherapy and untreated diabetic groups. In addition to our primary findings, the triple combination therapy prevented loss of body weight, protected against destruction of pancreatic islets and improved lipid profile.

### 4.1 Triple Combination Therapy Improved Glycemic Control Under Diabetic Condition

As glycosylated haemoglobin (HbA1c; a binding product of hemoglobin in erythrocytes and blood glucose) is the most reliable and widely accepted biomarker for evaluating glycemic control, we observed a 3-fold increase in HbA1c level with a corresponding weight loss in our T2DM rats, which are common hallmarks of untreated or uncontrolled diabetes mellitus, and places diabetic patients at increased risk of adverse cardiac events. Our finding supports that of a prospective observational study in which every 1% increase in HbA1c levels in T2DM patients was linked to 8% increase risk of developing DCM independent of traditional cardiovascular risk factors (Stratton et al., 2000) while every 1% increase in HbA1c levels in T1DM patients was also associated with 30% increase risk of heart failure (Lind et al., 2011). This suggests that hyperglycemia is a dominant factor that promotes adverse cardiac events in diabetic patients. There is also insulin resistance and inappropriate activation of systemic and tissue renin-angiotensin-aldosterone system (RAAS), which impair cardiac insulin metabolic signaling in DCM (Xu et al., 2013; Jia et al., 2015; Jia et al., 2016). Therefore, current treatment guidelines by various organizations for diabetic patients strongly emphasize close monitoring and strict glycemic control with anti-diabetic drugs and other pharmacological agents with the aim of maintaining normoglycemia and improving cardiac outcomes. In the present study, administration with gliclazide, a commonly used second-generation sulfonylurea for the management of T2DM, significantly reduced HbA1c level, albeit did not restore normoglycemia. A similar observation was made following monotherapy with ALA, although ALA had a better anti-hyperglycemic effect than gliclazide. Treatment with ramipril (a known angiotensin-converting enzyme inhibitor for treating cardiovascular diseases) did not have a significant impact on HbA1c level compared to monotherapies with gliclazide and ALA. However, triple combination therapy with ALA, gliclazide and ramipril prevented hyperglycemia and maintained normoglycemia in our T2DM rats.

### 4.2 Possible Glycemic Control Mechanism by Triple Combination Therapy

As in skeletal muscle and other tissues, transport of glucose in cardiac tissue is mediated by glucose transporter protein subtype 4 (GLUT 4). Translocation of GLUT4 to the cardiomyocyte plasma membrane is decreased in DCM due to impaired insulin metabolic signaling, resulting in decreased activity of sarcoplasmic reticulum Ca^2+^ pump and increasing cardiomyocyte intracellular Ca^2+^ concentration (Jia et al., 2016). Our observation with the triple combination therapy, which includes adequate protection of the pancreatic islets (implying preservation of insulin-releasing β-cells), suggests a synergistic effect and/or activation of different individual mechanisms by each of these drugs. Such synergism may have prevented or attenuated impaired insulin metabolism and possibly may have partly contributed to stimulating insulin release from functioning pancreatic β-cells via reduction of potassium ion permeability (a known mechanism of insulin secretion) and increasing peripheral tissue sensitivity to insulin, and thereby decreasing insulin resistance (Agrawal and Gupta, 2013). Several mechanisms have been implicated in the anti-hyperglycemic effect of ALA. For example, ALA has been reported to improve glycemic control through protection of pancreatic β-cells with preserved islet function (Cummings et al., 2010; Yi et al., 2011) and also possesses insulin-mimetic activity, enhancing the activity of insulin receptor, and thereby leading to cytoprotection via inhibition of apoptotic pathway (Diesel et al., 2007). It also increases insulin sensitivity in skeletal and cardiac muscles by recruiting GLUT4 to the plasma membrane and activating AMP-activated protein kinase (AMPK) signalling pathways in skeletal muscle and diabetic heart (Strödter et al., 1995; Khamaisi et al., 1997; Lee et al., 2005) as well as phosphoinositide-3-kinase (PI3K), promoting tyrosine phosphorylation in the insulin receptor and improving PI3K-dependent glucose uptake (Khamaisi et al., 1997; Yaworsky et al., 2000; Moini et al., 2002). All these mechanisms by ALA inhibit insulin resistance and facilitate glucose uptake and utilization by cells including cardiomyocytes, and thus enhancing sarcoplasmic reticulum Ca^2+^ pump activity and reducing cardiomyocyte intracellular Ca^2+^ concentration (55). These mechanisms of ALA together with the well-established closure of ATP-sensitive potassium channels (in β-cell plasma membrane) resulting in Ca^2+^ influx and Ca^2+^-dependent insulin granule exocytosis by sulfonylureas such as gliclazide (Lawrence et al., 2001; de Wet and Proks, 2015; Proks et al., 2018) and the mild glucose-lowering effect of ramipril on HbA1c, may account for the maintenance of normoglycemia observed following the triple combination therapy in the present study. Our result suggests that ALA could serve as an adjuvant to conventional anti-diabetic therapy for DCM. However, a major concern of our novel pharmacotherapeutic approach is the possible risk of hypoglycemia, which will therefore, necessitate further studies to determine this possibility and whether or not a dose adjustment would be needed for long-term glycemic control. Another important pharmacokinetic factor to consider in future studies is the route of ALA administration. Although our finding showed improved glycemic control following oral administration of ALA in both mono- and combined therapy groups, a clinical study in T2DM patients showed that intravenous administration of ALA increases insulin-mediated glucose uptake with improved glycemic control while oral administration had only marginal effects on diabetic complications (Ziegler et al., 1999). Overcoming this limitation of oral administration could make ALA a safe and effective adjuvant to existing anti-diabetic therapy with insulin-sensitizing activity while providing a potential advantage by allowing self-administration by diabetic patients.

### 4.3 Triple Combination Therapy Protected Against Development of Diabetic Cardiomyopathy

The focal point of the present study is our observation of marked degeneration and vacuolation of myocardial fibers in untreated diabetic rats, which positively correlated with elevated levels of cardiac damage markers such as troponin I (cTnI), creatine kinase myoglobin band (CK-MB) and brain natriuretic peptide (BNP) in plasma and heart tissue. cTnI and CK-MB are powerful biomarkers for sensitive and specific detection of cardiomyopathies such as DCM or cardiac injury from various causes while BNP is a non-specific but sensitive biomarker for predicting cardiac dysfunction including DCM (Atabek et al., 2004; Korraa et al., 2012; El-Saiedi et al., 2020; Ajiboye et al., 2021). Thus, increased levels of these biomarkers predict the risk of cardiomyocyte death and subsequent adverse cardiac events. Monotherapy in the present study reduced the elevated levels of these cardiac damage markers in diabetic animals, signifying some protection against DCM progression. However, triple combination therapy resulted in superior cardioprotection, as the levels of the cardiac damage markers decreased substantially to the levels in healthy control rats. This suggests that co-administration of anti-diabetic drug and blockade of RAAS with an ACE inhibitor (e.g., ramipril) along with ALA supplementation protects against the development of DCM beyond that offered by conventional therapy. Our results are consistent with those of previous preclinical and clinical studies in which ALA and ramipril administrations reduced the levels of these cardiac damage markers and prevented development and progression of DCM (Symeonides et al., 2007; Hegazy et al., 2013; Mehanna et al., 2017).

In addition to showing that triple combination therapy prevents the development of DCM, we investigated possible mechanisms of cardioprotection by these drugs. Systemic changes such as high triglycerides production and cholesterol secretion with reduced high-density lipoprotein (HDL) cholesterol are well-documented pathological factors that contribute to structural and functional changes (cardiac lipotoxicity) and altered Ca^2+^ homeostasis in myocardium (Fukuda et al., 2010; Kuramoto et al., 2014; Jaishy et al., 2015). These cellular events are due to hyperinsulinemia and increased free fatty acid accumulation and oxidation in insulin-resistant adipocytes and cardiomyocytes, which contribute to the development and progression of DCM, and could account for the diastolic dysfunction in 40–75% of diabetic patients without coronary artery disease (Shivalkar et al., 2006; Brooks et al., 2008; Fukuda et al., 2010; Kuramoto et al., 2014; Jaishy et al., 2015). In accord, our data show high levels of plasma triglycerides and total cholesterol and low plasma HDL level in the untreated T2DM rats, which were reversed to healthy control values following mono- and triple combination therapies. Although we did not examine the cellular events which may have resulted in the lipid-lowering effects of the treatments in the present study, it is likely that ALA and the other drugs may have inhibited the pathological cellular pathways, leading to improved lipid profile in the diabetic rats. Our observation is consistent with those of previous studies in which ALA effectively lowered serum triglycerides, total cholesterol, low-density lipoprotein (LDL) and very-low-density lipoprotein (VLDL) cholesterol, and significantly increased serum HDL level in hypercaloric T2DM rats (Ghelani et al., 2017). The lipid-lowering effect of ALA, for example, is reported to be *via* reduced hepatic synthesis of 3-hydroxy-3-methylglutaryl coenzyme A reductase (HMG-CoA reductase; rate-limiting enzyme in cholesterol synthesis) (Rideout et al., 2016). As hepatic cholesterol catabolism is dependent on bile acid synthesis, ALA was found to also reduce bile acid synthesis *via* downregulation of CYP7A1 gene (the protein-coding gene of the rate-limiting enzyme in bile acid biosynthesis), and increased cholesterol clearance via reduced PCSK9 gene (protein that regulates LDL receptors) (Rideout et al., 2016). Such pleiotropic mechanisms of ALA, together with anti-lipemic effect of gliclazide (Emral et al., 2005; Pan et al., 2020) suggests that it can serve as an important complementary therapy not only in DCM but also in other cardiovascular conditions involving dyslipidemia.

### 4.4 Mechanisms Underlying Cardioprotection by Triple Combination Therapy Under Diabetic Condition

To assess further underlying mechanisms, we also observed increased oxidative stress, inflammation and fibrosis in plasma and cardiac tissue of untreated T2DM rats. Mitochondria and RAAS-mediated nicotinamide adenine dinucleotide phosphate (NADPH) oxidase as well as activation and expression of pro-inflammatory cytokines are principal sources of cardiac oxidative stress and fibrosis through increased ROS production and activation of TGF-β1/Smad signaling pathway, and contribute to DCM development and progression (Midaoui et al., 2003; Li et al., 2009; Lee et al., 2012; Murdoch et al., 2014; Jia et al., 2016). Our study showed prevention of oxidative stress following triple combination therapy. This was characterized by significantly decreased plasma and cardiac malondialdehyde (MDA; a by-product of lipid peroxidation and indication of ROS production) and increased cardiac content of the antioxidant enzymes glutathione (GSH) and superoxide dismutase (SOD) activity. Plasma levels of pro-inflammatory cytokines (e.g., TNF-α, IL-1β and IL-6) were also decreased to healthy control levels. Our western blot analysis of TGF-β1/Smad2 and 3 signaling pathway (a well-identified central mediator of cardiac fibrosis) also revealed markedly increased expression levels of TGF-β1 and phosphorylated Smad2 and 3 in the cardiac tissue of untreated diabetic rats, indicating activation of the TGF-β1/Smad signaling pathway. However, this fibrotic pathway was significantly inhibited following triple combination therapy, all of which partly contributed to cardiac protection and prevention of DCM in the T2DM rats. Whereas RAAS inhibition with ramipril treatment was previously reported to significantly reduce active TGF-β1/Smad2 and 3 protein levels in cardiac tissue of diabetic rats (Castoldi et al., 2009), we observed only a marginal decrease in the expression levels of these proteins in the myocardial tissue of our diabetic rats following monotherapy with ramipril. However, our triple combination therapy substantially decreased the expression levels of these fibrotic proteins to levels even below those of healthy control rats. This difference could be due to differences in the duration of treatment, as Castoldi et al. (2009) administered ramipril for 8 weeks while we administered it for 6 weeks. Also, our triple combination therapy may have produced synergism, resulting in the substantial inhibition of the TGF-β1/Smad2 and 3 signaling pathway.

As ROS-induced oxidative stress is the recognized unifying pathogenetic factor and a major molecular pathway in the development and progression of DCM and other macrovascular diabetic complications via several pathways including hyperglycemia, protein kinase C activation, formation of advanced glycation end-product, flux of polyol and hexosamine pathways, activation of nuclear factor kappa B (NF-κB; an inflammation-relevant transcription factor), our findings suggest that, as an antioxidant, ALA activates or recruits other antioxidant enzymes to boost the body’s antioxidant defense system against oxidative stress and its associated deleterious effects. Besides, gliclazide and ramipril as constituents of our triple combination therapy, reduced mitochondrial superoxide generation, inhibited NADPH oxidase (NOX) via azabicyclo-octyl ring structure of gliclazide, leading to suppressed intercellular adhesion molecule-1 (ICAM-1; an inflammatory glycoprotein), reduced ROS-induced oxidative stress, myocardial fibrosis, cardiomyocyte apoptosis, cardiomyocyte hypertrophy, diastolic dysfunction, and enhanced production of SOD in experimental models of DCM and diabetic nephropathy (Onozato et al., 2004; Huynh et al., 2012; Pan et al., 2020). Gliclazide also inhibited endoplasmic reticulum (ER) stress via downregulation of the ER stress proteins GRP78 and sXBP1 and mRNA expression, culminating in renal protection in experimental diabetic nephropathy (Zhang Y. W. et al., 2018). This mechanism of gliclazide may have produced the same salutary effect in DCM although it will require further investigation. The strong antioxidant effect of our combination therapy may further suggest that these drugs effectively inhibit hyperpolarization of the mitochondrial inner membrane and activate electron transport in complex III, leading to reduced ROS production. Our data also support an observation in a randomized control trial and other rat models of DCM in which ALA administration prevented an increase in heart mitochondrial ROS production and effectively enhanced SOD activity and GSH content of myocardial mitochondria as well as decreased collagen deposition and TGF-β1 and mitochondria-dependent cardiac apoptosis (Midaoui et al., 2003; Li et al., 2009; Lee J. E. et al., 2012; Hegazy et al., 2013). ROS promotes development of cardiac fibrosis in DCM by upregulating TGF-β1 and its downstream proteins Smad2 and 3 (Purnomo et al., 2013; Wang et al., 2019; Wang et al., 2021) as was also observed in the present study. Moreover, Micheloudes et al. (2011) reported that overexpression of TGF-β1 resulted in increased ROS-induced oxidative stress via increased expression of NADPH oxidase 4 (NOX4; a major modulator of ROS-related fibrosis) and decreased the activity of the mitochondrial antioxidant manganese-dependent superoxide dismutase (MnSOD). Also, deletion of Smad3 gene decreased cardiomyocyte hypertrophy and myocardial fibrosis along with reduced myocardial oxidative stress, leading to improved cardiac compliance in mice (Biernacka et al., 2015). In addition, Smad7 protein, which is well-known to inhibit phosphorylation of Smad2 and 3, inhibited NADPH-mediated ROS generation and prevented cardiac fibrosis (Yu et al., 2013), implying that inhibition of Smad2 and 3 phosphorylation prevents activation of the TGF-β1/Smad pathway, and thereby protecting against cardiac fibrosis. These pieces of empirical evidence suggest that a crosstalk exists between ROS-induced oxidative stress and fibrotic factors.

### 4.5 Limitations of the Study

On the other hand, our novel pharmacotherapeutic approach has a number of limitations. Although we measured blood glucose levels and performed pancreas histology, which revealed destruction of the pancreatic islets in untreated diabetic rats, and was preserved following the triple combination therapy, we were unable to measure blood insulin levels due to technical challenges. Measurement of blood insulin would have given an additional information and a more comprehensive understanding of the effect of monotherapy versus triple combination therapy on insulin secretion. However, based on the pancreas histopathological images and HbA1c levels in the present study, we can predict that blood insulin level would be significantly decreased in the untreated diabetic control rats and possibly within the range of healthy control values in rats which received the triple combination therapy. We were also unable to stain for mononuclear inflammatory cells infiltrating the cardiac tissue, which would have given a broader picture of the degree of inflammation occurring in ventricles of the heart during DCM. In addition, we could not perform pressure-volume loop analysis and echocardiography and other parameters of myocardial function, which could have displayed the association between T2DM and end-systolic diameters, global systolic function and diastolic function through transmittal Doppler velocity profile. Furthermore, our Western blot analysis did not include Smad 7 protein, which could have provided additional information on phosphorylation of Smad2 and 3 proteins and activation of the TGF-β1/Smad signaling pathway in DCM and upon pharmacological therapy.

Despite the above limitations, our study is the first to show that triple combination therapy of ALA, gliclazide and ramipril prevents early development and progression of DCM in T2DM through inhibition of ROS-dependent TGF-β1/Smad pathway. Hence, ALA could emerge as a safe and effective adjunctive anti-diabetic therapy with insulin-sensitizing activity along with angiotensin-converting enzyme inhibitors such as ramipril for prevention and treatment of DCM. Our findings provide important addition to the existing body knowledge about DCM and its pharmacological treatment and management. However, much remains unknown. Thus, our findings can be extrapolated to the human heart to gain more insight into the pathophysiology of human DCM and its pharmacological therapy.

## Data Availability

The original contributions presented in the study are included in the article/Supplementary Material, further inquiries can be directed to the corresponding author.
