# Supplementary figures and images for: Combination Therapy of Alpha-Lipoic Acid, Gliclazide and Ramipril Protects Against Development of Diabetic Cardiomyopathy via Inhibition of TGF-β/Smad Pathway
Source: Front Pharmacol. 2022 Mar 21;13:850542. doi: 10.3389/fphar.2022.850542 (PMC8988231; doi:10.3389/fphar.2022.850542)

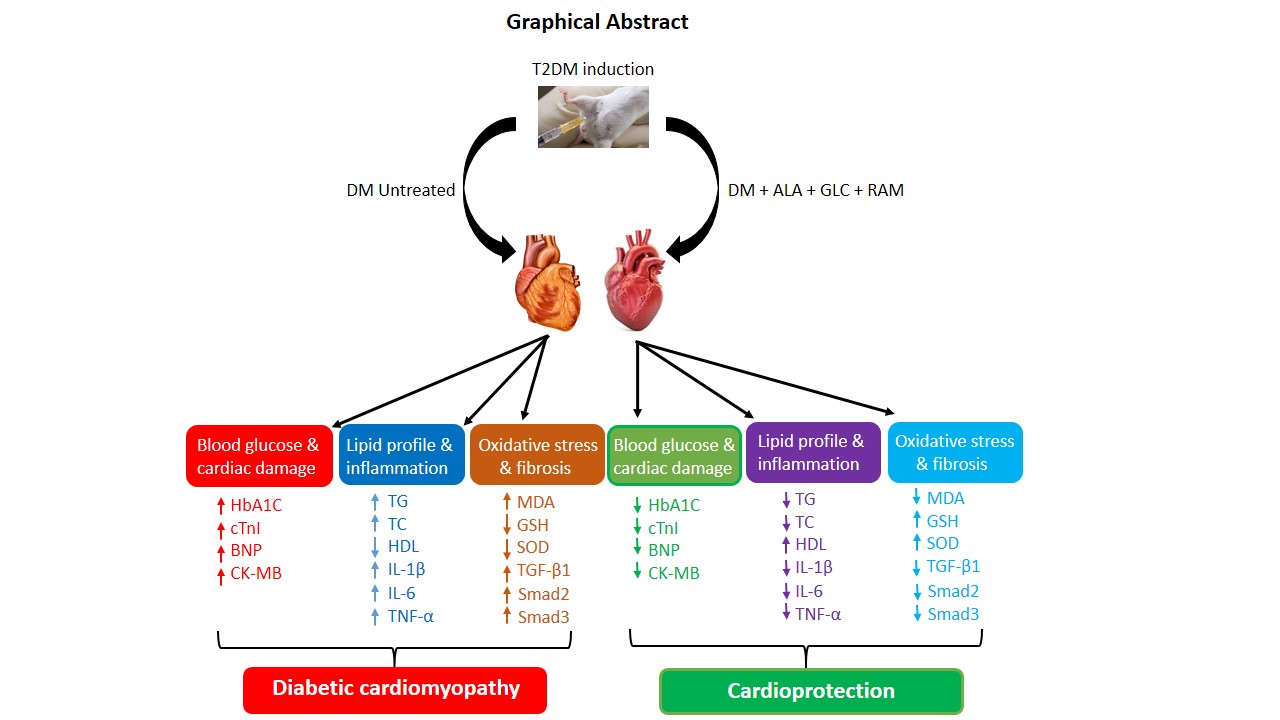

Supplement: Supplementary file 1 [file Image1.JPEG]
